# Draft Genome Sequences of Nine *Pseudomonas aeruginosa* Strains, Including Eight Clinical Isolates

**DOI:** 10.1128/genomeA.01154-15

**Published:** 2015-10-08

**Authors:** Patricio Jeraldo, Scott A. Cunningham, Daniel Quest, Robert A. Sikkink, Daniel O’Brien, Bruce W. Eckloff, Robin Patel, Nicholas Chia

**Affiliations:** a Department of Surgery, Mayo Clinic, Rochester, Minnesota, USA; b Center for Individualized Medicine, Mayo Clinic, Rochester, Minnesota, USA; c Carl R. Woese Institute for Genomic Biology, University of Illinois, Urbana, Illinois, USA; d Department of Laboratory Medicine and Pathology, Division of Clinical Microbiology, Mayo Clinic, Rochester, Minnesota, USA; e Medical Genomics Facility, Mayo Clinic, Rochester, Minnesota, USA; f Department of Health Sciences Research, Mayo Clinic, Rochester, Minnesota, USA; g Department of Medicine, Division of Infectious Diseases, Mayo Clinic, Rochester, Minnesota, USA

## Abstract

We report on nine draft genomes of *Pseudomonas aeruginosa* isolates, assembled using a hybrid paired-end and Nextera mate-pair library approach. Eight are of clinical origin, and one is the ATCC 27853 strain. We also report their multilocus sequence types.

## GENOME ANNOUNCEMENT

*Pseudomonas aeruginosa* is an important pathogen, associated with infections in burn wounds, ventilated patients, patients with cystic fibrosis, and so on. It is intrinsically resistant to a number of antimicrobial agents and may acquire resistance to any of the available antimicrobics. Further, *P. aeruginosa* may be nosocomially transmitted. The complete genome sequences of eight clinical isolates from a single facility were obtained for epidemiological studies of relatedness (i.e., clonality). ATCC 27853 was also studied.

Isolates were cultivated on 5% sheep blood agar and genomic DNA obtained using the Maxwell 16 Tissue DNA purification kit (Promega, Madison, WI, USA) and the Genomic DNA Clean and Concentrator kit (Zymo Research, Irvine, CA, USA).

Paired-end and two Nextera mate-pair DNA libraries from each cultured isolate were prepared with target fragment lengths of 500 bp for the paired-end reads, and 4 to 6 kbp, and 8 to 10 kbp for the mate-pair reads, respectively. A total of 60,176,290 read pairs were produced on the Illumina MiSeq platform run for 600 cycles across all nine genomes. Reads were processed for adapter removal and quality filtering using Trimmomatic version 0.32 (1) with parameters “ILLUMINACLIP:adapters.fasta:2:30:10 LEADING:3 TRAILING:3 MAXINFO:220:0.1 MINLEN:70.” Reads were then error corrected using SPAdes version 3.1.0 (2) in error-correction-only mode. Read coverage was decreased to 80× by randomly choosing read pairs. Reads were then assembled in two passes. First pass assembly was performed using Velvet version 1/2/10 (3) with *k*-mers from 31 to 99, selecting contigs from the assembly with the longest contig. The final pass assembly was performed using SPAdes in assembly-only mode, using as input the same unassembled reads as the first pass assembly, plus the contigs from Velvet passed as “untrusted contigs” to SPAdes. For each isolate, we identified the genes used for multilocus sequence typing of *P. aeruginosa*, and submitted them for typing using the PubMLST (4) database (http://pubmlst.org/paeruginosa). The resulting genomes, their multilocus sequence types (MLSTs), and assembly statistics are listed in Table 1.

**TABLE 1:** List of accession numbers, sequence types, and assembly statistics for the genomes submitted under this announcement

Isolate	Accession no.	MLST	No. of contigs (>300 bp)	G+C content (%)	Assembled length (bp)	*N*_50_ length (bp)
ATCC 27853	LFMN00000000	ST-155	21	66.08	6,847,745	2,097,247
BTP031	LFMO00000000	ST-235	15	65.98	6,878,781	1,039,251
BTP032	LFMP00000000	ST-235	32	66.11	7,547,174	2,492,409
BTP033	LFMQ00000000	ST-235	22	65.95	6,899,762	1,220,050
BTP034	LFMR00000000	ST-654	16	65.99	6,924,697	1,036,494
BTP035	LFMS00000000	ST-235	19	65.97	6,873,380	2,067,829
BTP036	LFMT00000000	ST-235	1	65.98	6,879,005	6,879,005
BTP037	LFMU00000000	ST-235	44	65.99	6,857,129	5,465,220
BTP038	LFMV00000000	ST-823	41	66.00	7,085,577	1,371,882

### Nucleotide sequence accession numbers.

The complete genome sequences have been deposited at DDBJ/EMBL/GenBank under the accession numbers provided in Table 1.
